# Transcript levels of *orf288* are associated with the *hau* cytoplasmic male sterility system and altered nuclear gene expression in *Brassica juncea*

**DOI:** 10.1093/jxb/erx443

**Published:** 2018-01-02

**Authors:** Shuangping Heng, Jie Gao, Chao Wei, Fengyi Chen, Xianwen Li, Jing Wen, Bin Yi, Chaozhi Ma, Jinxing Tu, Tingdong Fu, Jinxiong Shen

**Affiliations:** 1 National Key Laboratory of Crop Genetic Improvement, College of Plant Science and Technology, National Center of Rapeseed Improvement in Wuhan, Huazhong Agricultural University, Wuhan, P.R. China; 2 College of Life Science, Institute for Conservation and Utilization of Agro-Bioresources in Dabie Mountains, Xinyang Normal University, Xinyang, P.R. China

**Keywords:** Anther development, *Brassica*, cytoplasmic male sterility, *hau*, CMS, *orf288*, plant mitochondria

## Abstract

Cytoplasmic male sterility (CMS) is primarily caused by chimeric genes located in the mitochondrial genomes. In *Brassica juncea*, *orf288* has been identified as a CMS-associated gene in the *hau* CMS line; however, neither the specific abortive stage nor the molecular function of the gene have been determined. We therefore characterized the *hau* CMS line, and found that defective mitochondria affect the development of archesporial cells during the L2 stage, leading to male sterility. The expression level of the *orf288* transcript was higher in the male-sterility line than in the fertility-restorer line, although no significant differences were apparent at the protein level. The toxicity region of ORF288 was found to be located near the N-terminus and repressed growth of *Escherichia coli*. However, transgenic expression of different portions of ORF288 indicated that the region that causes male sterility resides between amino acids 73 and 288, the expression of which in *E. coli* did not result in growth inhibition. Transcriptome analysis revealed a wide range of genes involved in anther development and mitochondrial function that were differentially expressed in the *hau* CMS line. This study provides new insights into the *hau* CMS mechanism by which *orf288* affects the fertility of *Brassica juncea.*

## Teaser

High transcript levels of the CMS-associated gene *orf288* in the *hau* CMS line of *Brassica juncea* induce defects in mitochondrial ultrastructure during the development of archesporial cells and cause cytoplasmic male sterility.

## Introduction

Cytoplasmic male sterility (CMS) is primarily caused by chimeric genes located in the mitochondrial genome, and it can be suppressed by restoration-of-fertility (*Rf*) genes in the nuclear genome (Schnable, 1998; Chase, 2007). This system is not only a useful method for hybrid seed production, but also serves as a model for investigating nuclear–mitochondrial interactions (Hanson and Bentolila, 2004). Various CMS systems have been identified, and they are widely used in different crop plants, including maize, rice, wheat, petunia, pepper, and *Brassica* species (Li *et al.*, 2007; Chen and Liu, 2014). Importantly, specific CMS mechanisms are often completely distinct in different plants. To date, four models have been proposed to explain CMS mechanisms, namely the cytotoxicity model, the energy deficiency model, the aberrant programmed cell death (PCD) model, and the retrograde regulation model (Chen and Liu, 2014). Although some CMS-associated genes can suppress the growth of prokaryotic cells (Korth *et al.*, 1991; Nakai *et al.*, 1995; Duroc *et al.*, 2005; Wang *et al.*, 2006; Jing *et al.*, 2012), direct evidence for CMS proteins mediating cytotoxicity in plants is still lacking. In the energy deficiency model, CMS proteins cause male sterility by disrupting normal mitochondrial energy requirements during male reproductive development (Ducos *et al.*, 2001; Ji *et al.*, 2013; Wang *et al.*, 2013). Premature tapetal PCD has been observed in the CMS-PET1 cytoplasm in sunflower (Balk and Leaver, 2001) and CMS-WA (wild-abortive-type) cytoplasm in rice (Luo *et al.*, 2013), which supports the aberrant PCD model. Mitochondrial CMS genes can also regulate the expression of nuclear genes involved in anther development through retrograde signaling, consistent with the retrograde regulation model. In plants with Chinese wild rice-type CMS, overexpression of the *RMS* (*RETROGRADE-REGULATED MALE STERILITY*) gene in a fertility restorer line caused male sterility, whereas RNAi suppression of *RMS* restored fertility to CMS plants (Fujii and Toriyama, 2009). Although CMS has been extensively exploited in hybrid seed production in crop species, the molecular mechanisms underlying differential male sterility remain elusive.

Numerous different CMS systems have been reported in Brassicaceae, including among others *nap* CMS, *pol* CMS, *ogu* CMS, *Tour* CMS, *Moricandia arvensis* CMS, *Nsa* CMS, *NCa* CMS, *hau* CMS, and *inap* CMS (Grewe *et al.*, 2014; Heng *et al.*, 2015; Yang *et al.*, 2016). However, only a few CMS-associated genes have been reported. In *B. napus*, the transcript expression level of the *pol* CMS-associated gene *orf224* was found to be higher in the male-sterile line than in the male-fertile line (L’Homme and Brown, 1993; Menassa *et al.*, 1999; Liu *et al.*, 2016). Expression of the *orf222*/*nad5c*/*orf139* region may be associated with *nap* CMS (L’Homme *et al.*, 1997; Liu *et al.*, 2017). ORF138 is a mitochondrial protein that acts at the inner mitochondrial membrane pore (Duroc *et al.*, 2009) and is responsible for *ogura* CMS in Brassiceae (Grelon *et al.*, 1994; Duroc *et al.*, 2005). Transcription of the *Kosena* CMS-associated gene *orf125*, which is homologous to *orf138*, is strongly associated with the CMS phenotype in *B. napus* (Landgren *et al.*, 1996). Studies on mitochondrial RNA and protein banding patterns revealed that *orf263* is associated with CMS in *B. tournefortii* cytoplasm (Landgren *et al.*, 1996). The *orf108* gene is also associated with CMS in the cytoplasm of *Moricandia arvensis* (Ashutosh *et al.*, 2008; Kumar *et al.*, 2012). A chimeric *orf220* gene from CMS *B. juncea* (stem mustard) causes male sterility (Yang *et al.*, 2010) and *MSH1-RNAi* from spontaneous fertile-revertant lines was found to increase the *orf220* copy number and induce male sterility (Zhao *et al.*, 2016).

Flowers are one of the most complex plant structures, and anther development in particular is orchestrated by complex gene regulatory networks (Pearce *et al.*, 2015). Molecular genetic studies have revealed many crucial genes involved in early anther development. For example, *WUSCHEL* (*WUS*) is critical for stem-cell fate determination in the shoot apical meristem of higher plants (Su *et al.*, 2009). In Arabidopsis, *SPL/NZZ*, which belongs to the MADS-box transcription factor family (Liu *et al.*, 2009), regulates anther patterns and sporocyte development. Moreover, *spl/nzz* mutants do not have microsporocytes and do not form an anther somatic wall layer (Schiefthaler *et al.*, 1999; Yang *et al.*, 1999). *AP3* and *PI*, which belong to the floral homeotic B function MADS-box gene family, determine petal and stamen development and orchestration (Jack *et al.*, 1992; Goto and Meyerowitz, 1994). *EMS1* encodes a leucine-rich repeat receptor protein kinase that controls somatic and reproductive cell fates in the Arabidopsis anther (Zhao *et al.*, 2002). *DYT1*, which encodes a putative bHLH transcription factor, controls anther development (Zhang *et al.*, 2006). To date, many genes and regulation networks involved in anther development and cell fate specification have been reported (Gómez *et al.*, 2015). Microarray and RNA-Seq have been widely used to analyse essential genes involved in *B. juncea* anther development. A number of mitochondrial genes and those involved early anther development (e.g. *WUS*, *SPL*, *AP3*, *PI*, *DYT1*, and *AMS*) were found to be down-regulated in male-sterility lines (Yang *et al.*, 2010; Zhao *et al.*, 2016).

Previous studies have identified *hau* CMS as being a novel CMS system in *B. juncea*, and the cytoplasm has been transferred to *B. napus* (Wan *et al.*, 2008) and *B. rapa* (Heng *et al.*, 2015). The *orf288* gene is responsible for male sterility in *hau* CMS in *B. juncea* (Jing *et al.*, 2012). After comparative analysis of the *hau* CMS mitochondrial genome and other sequenced mitochondrial genomes, the cytoplasm of *hau* CMS was identified as an alloplasmic cytoplasm in *Brassica* crops (Heng *et al.*, 2014). However, the specific stage of anther development that is affected and how the CMS-associated gene *orf288* triggers male sterility are poorly understood. Here, we describe the *hau* CMS line in *B. juncea*, which aborts at the archesporial cell stage of anther development. The *hau* CMS-associated gene *orf288* was expressed at higher levels in anthers from the *hau* CMS line than in the fertility-restorer line. We also found that the genetic region of *orf288* that inhibits the growth of *E. coli* is not associated with the *hau* CMS phenotype. Finally, expression of the *orf288* transcript affects anther development in other *Brassica* species.

## Materials and methods

### Plant material

The *hau* CMS line (6-102A) and its iso-nuclear maintainer line (6-102B) in *Brassica juncea* (Wan *et al.*, 2008) and the *hau* CMS line, its iso-nuclear maintainer line, and the fertility-restorer line in *B. napus* were grown at the rapeseed research field site at Huazhong Agricultural University (Wuhan, China) in September 2014. Wild-type (WT) *Arabidopsis thaliana* Columbia (Col-0) plants were grown under white fluorescent light (16 h light/8 h dark) at 22 °C during the day and 18 °C at night, with a relative humidity of 50%.

### Histological analyses

Scanning electron microscopy (SEM) was used to examine the surfaces of 6-102A and 6-102B anthers. Fresh anthers <1 mm in length were fixed overnight in 2% glutaraldehyde. The dehydrated samples were sputter-coated with gold and examined using a LEO 435VP scanning electron microscope (LEO Electron Microscopy Ltd). Fresh anthers from 6-102A and 6-102B at different developmental stages were fixed in 2.5% (w/v) glutaraldehyde in 0.2 M phosphate buffer (pH 7.2) for transmission electron microscopy (TEM) analysis. The procedures were performed as previously described (Yi *et al.*, 2010). Ultra-thin sections were obtained by using a Leica UC6 ultramicrotome and stained with uranyl acetate and lead citrate. A Hitachi H-7650 transmission electron microscope was used to record the images. Semi-thin sections were used to examine the *hau* CMS abortive stage, as described previously (Dun *et al.*, 2011). Flower buds from 6-102A and 6-102B were fixed and vacuum-treated overnight in FAA solution (alcohol: acetic acid: formalin: water ,10:1:2:7). A graded ethanol series (70, 85, 95, and 100%) was used to dehydrate the fixed anthers. The anthers were then embedded in Technovit 7100 (Heraeus) resin for the semi-thin section analysis. The samples were cut into approximately 2-μm thick sections, stained with Toluidine Blue (Sigma-Aldrich), and photographed using bright-field microscopy.

### Northern and western blotting

RNA was isolated from the top floral buds (<0.5 mm) from the *hau* CMS line in *B. juncea* and *B. napus*, its maintainer line in *B. napus*, and floral buds of different sizes (<0.5 mm; 0.5–2 mm; 2–4 mm) from the fertility-restorer line in *B. napus* in April 2015. Northern blotting analysis was performed as previously described (Jing *et al.*, 2012) to detect the *orf288* expression patterns in the *hau* CMS line and its fertility-restorer line. Total RNA from different samples was fractionated on a 1.2% denaturing agarose gel containing 2% formaldehyde and transferred to Hybond N^+^ membranes (Amersham, UK). The *orf288* and *atp6* expression levels were detected using a Promega labeling kit and HYB-101 Perfect Hyb (ToYoBo). Membranes were exposed on a phosphor storage screen for 2 h, and the signals were scanned using a Typhoon FLA 9000 imaging system (Fujifilm, Japan).

Total protein was also extracted from floral buds (<0.5 mm) from the *hau* CMS line in *B. juncea* and *B. napus*, its maintainer line, and the fertility-restorer line in *B. napus* in April 2015. Proteins were separated using 10% SDS–PAGE or Tricine gels and transferred to a PVDF membrane for western blotting (Millipore). A peptide antigen corresponding to 150 residues of ORF288 was synthesized using a chemical synthesis method (ABclonal) and then used to immunize rabbits for antibody production. An anti-β-actin antibody was used as a positive control.

### Expression of different truncated *orf288* transcripts in *E. coli*

Full-length and truncated *orf288* fragments were amplified from flower bud cDNAs (primers are listed in Table S1 at the Dryad Digital Repository, https://doi.org/10.5061/dryad.9s68p; the BamHI and HindIII restriction sites in the primer sequences are underlined). Six different truncated *orf288* fragments were amplified (see Results) and cloned into the PET32a bacterial expression vector (Novagen). When the optical density (OD) of the samples reached 0.6, isopropyl-β-D-thiogalactopyranoside (IPTG; 0.5 mM) was added to induce expression of the full-length and truncated ORF288 fragments in *E. coli* BL21 (DE3) plysS cells (Promega). The OD of each sample was measured every 30 min at 600 nm using a UV-1601 spectrophotometer (Shimadzu, Japan) five times with three replicates each.

### Plant expression vector construction and transformation complementation test

A DNA fragment containing a mitochondrial targeting peptide (159 bp of the restorer gene *Rfp*) and the full-length or truncated ORF288 sequences were cloned into the pCAMBIA2300 binary vector under a double CaMV 35S promoter (Fig. S1 at Dryad). The *Agrobacterium tumefaciens* (GV3101)-mediated floral dip transformation method was performed in *A. thaliana* (Clough and Bent, 1998). The transgenic plants were analysed by PCR with primers specific to the CMS-associated genes (primers are listed in Table S1 at Dryad).

### RNA sequencing and data processing

The RNAprep Pure Plant Kit (TIANGEN DP441) was used to extract total RNA from the different floral buds (<0.5 mm) from 6-102A and 6-102B, with three replications each. Sequencing was performed using the Illumina NextSeq 500 platform. Tophat2 was used to align the RNA-Seq reads against the *B. juncea* genome (http://brassicadb.org). HTSeq 0.6.1p2 was used to calculate the read counts for each gene. The reads per kilobase per million mapped reads (RPKM) value was used to estimate the expression levels of the different genes. Genes with a fold change >2 and a *P*-value <0.05 were identified as differentially expressed genes (DEGs) when using DESeq (v1.16) for the gene expression analysis.

First-strand cDNA was synthesized using the QuantiTect Reverse Transcription Kit according to the manufacturer’s instructions. Quantitative real time PCR (qRT-PCR) was performed using the SYBR Green Real-time PCR Master Mix (TOYOBO, Japan) on a Bio-Rad CFX96 instrument. PCRs had the following cycling conditions: (1) 95 °C for 5 min; (2) 40 cycles at 95 °C for 10 s, 60 °C for 15 s, and 72 °C for 30 s; and (3) a final extension step at 72 °C for 5 min. Results were analysed using the CFX Manager software program according to the 2^−ΔΔ*C*T^ method (Livak and Schmittgen, 2001). The primers used for qRT-PCR are listed in Table S1 at Dryad.

## Results

### Flower morphological defects in the *hau* CMS line in *B. juncea*

**Fig. 1.**
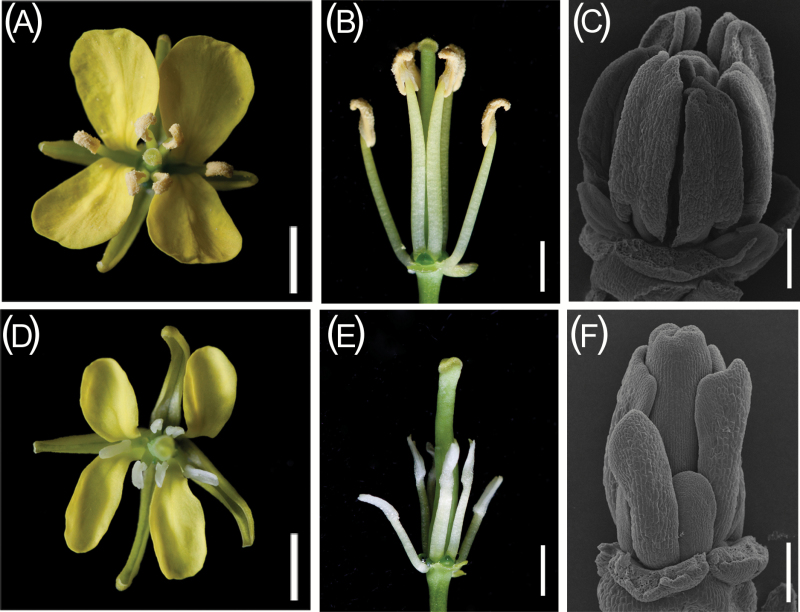
Anther phenotypes in the *hau* CMS lines and the maintainer line in *B. juncea*. (A, B) A flower from the *hau* CMS maintainer line (6-102B). (D, E) A flower from the *hau* CMS line (6-102A). Scale bars =20 mm. (C, F) A mature anther from the *hau* CMS maintainer line (C) and the *hau* CMS line (F). Scale bars =200 µm. (This figure is available in colour at *JXB* online.)

**Fig. 2.**
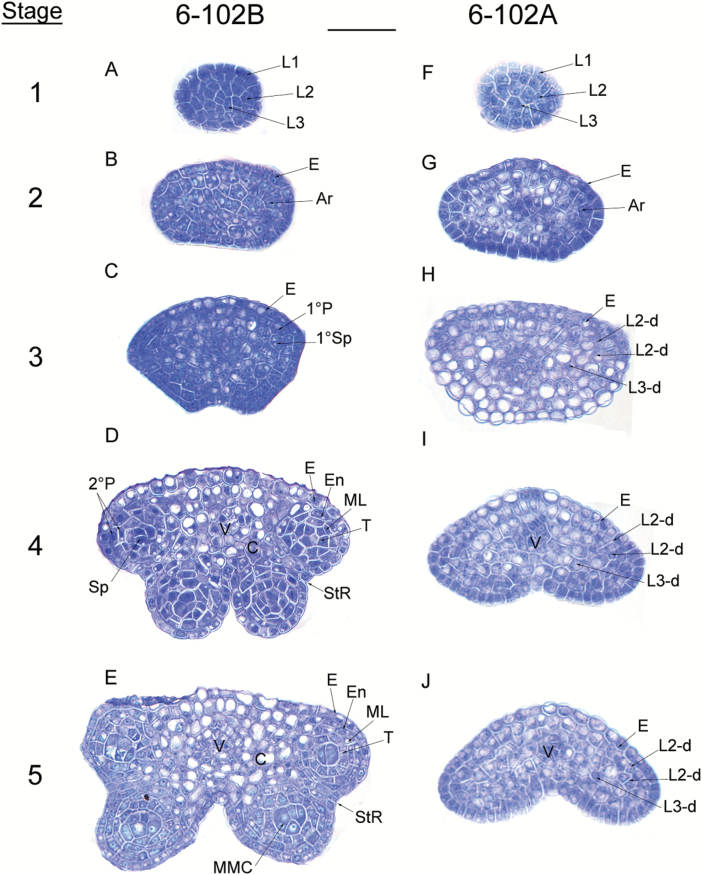
Anther development defects in the *hau* CMS line in *B. juncea*. The images are semi-thin sections from (A–E) the *hau* CMS maintainer line (6-102B) and (F–J) the *hau* CMS line (6-102A) showing anther development from stages 1–5. Abbreviations: Ar, archesporial cell; E, epidermis; En, endothecium; L1, L2, and L3, the three cell layers of the stamen primordia; L2-d and L3-d, the L2- and L3-derived cells; ML, middle layer; MMC, microspore mother cell; Sp, sporogenous cells; StR, stomium region; T, tapetum; V, vascular tissue; 1°P, primary parietal layer; 1°Sp, primary sporogenous layer; and 2°P, secondary parietal cell layers. Scale bar =25 µm. (This figure is available in colour at *JXB* online.)

The morphological features of 6-102A (*hau* CMS) and 6-102B (*hau* CMS maintainer) were compared. Petals from 6-102A were reduced in size compared with those from 6-102B (Fig. 1D), And short filaments were also observed in flowers from the *hau* CMS line (Fig. 1E). The stamens of the *hau* CMS line were transformed into thickened petal-like structures (Fig. 1D, E). SEM results revealed that the petal-like stamens in the *hau* CMS line formed at an early stage of flower development (Fig. 1F). No differences in vegetative growth were observed between the plants. To better understand which cell types were affected in the stamens of the *hau* CMS line, transverse sections of 6-102A and 6-102B anthers at different stages of development were examined. Anther development can be divided into 14 stages in Arabidopsis (Sanders *et al.*, 1999), and similar developmental stages have been described in *B. juncea*. Anther development was similar in the *hau* CMS line and its iso-nuclear maintainer line before the differentiation of archesporial cells (Fig. 2A, F, and B, G). Archesporial cells in 6-102B anthers underwent asymmetric and symmetrical cell divisions and differentiated into the endothecium, middle layer, tapetum, and microspore mother cells during stages 2–5. However, in an effect that was derived from the L2 stage of development, cell differentiation stopped and the divisions of each layer did not generate the secondary parietal layers and sporogenous cells in 6-102A (Fig. 2H). Anthers from the *hau* CMS line were completely aborted, with no pollen sacs (Fig. 2 H– J). Based on these cytological observations, we concluded that the archesporial cells arrest differentiation at stage 2, leading to abnormal stamens and male sterility in the 6-102A plants.

### Defective mitochondria in the anther cause male sterility in the *hau* CMS line

**Fig. 3.**
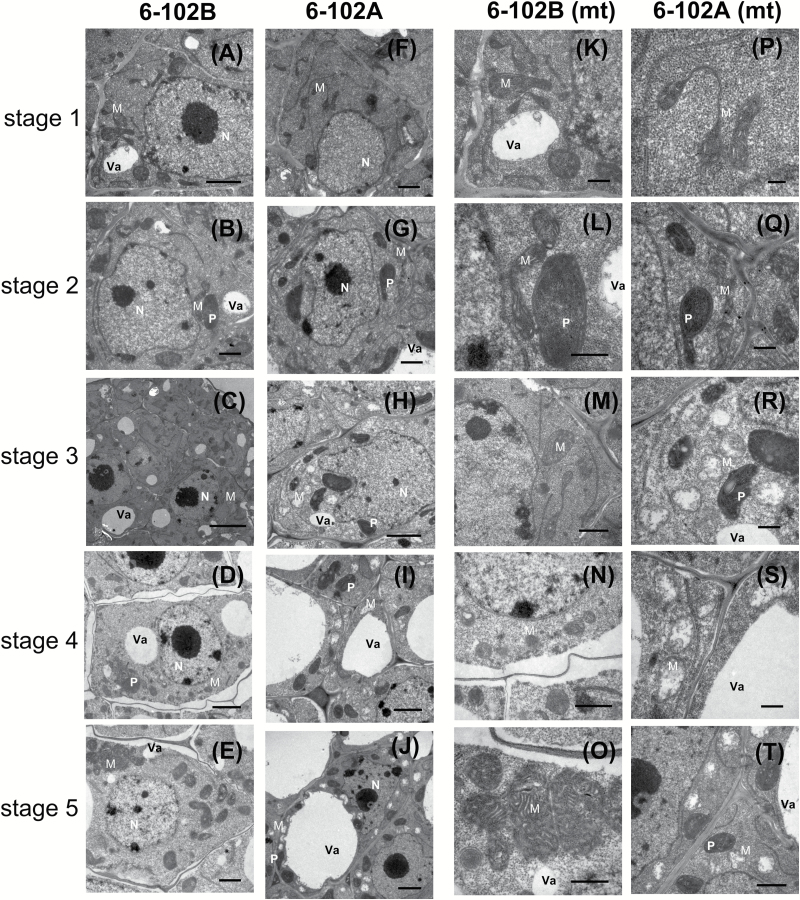
TEM analysis of anthers from the *hau* CMS maintainer line (6-102B) and the *hau* CMS line (6-102A) at different stages. Anther development from stages 1–5 in 6-102B (A–E) and in 6-102A (F–J). The mitochondrial structures of the cells from (A–E) and (F–J) are shown in (K–O) and (P–T), respectively. Abbreviations: M, mitochondria; P, plastid; N, nucleus; and Va, vacuole. Scale bars: (D) 0.2 µm, (B, F, H, L, P, R) 0.5 µm, (C, E, G, J, N, Q, T) 1 µm, (A, K, M, O, S) 2 µm, and (I) 5 µm.

Notably, CMS systems in different plants are primarily caused by mitochondrial disturbances in the anther. TEM was used to compare the cell structures between 6-102A and 6-102B, and no significant differences were observed prior to stage 2 of anther development. The mitochondria were integrated with a double-membrane structure, and mitochondrial crests were clearly visible (Fig. 3F, G, P, Q). By contrast, numerous vacuolated mitochondria could be seen in sterile male anthers with swollen cells after stage 3 (Fig. 3R–T). More defective mitochondria were present in cells with sterile anthers. These observations suggest that *orf288* affects mitochondrial function and interrupts tissue differentiation in anthers from the *hau* CMS line, leading to male sterility.

### Expression profile of the CMS-associated gene *orf288*

**Fig. 4.**
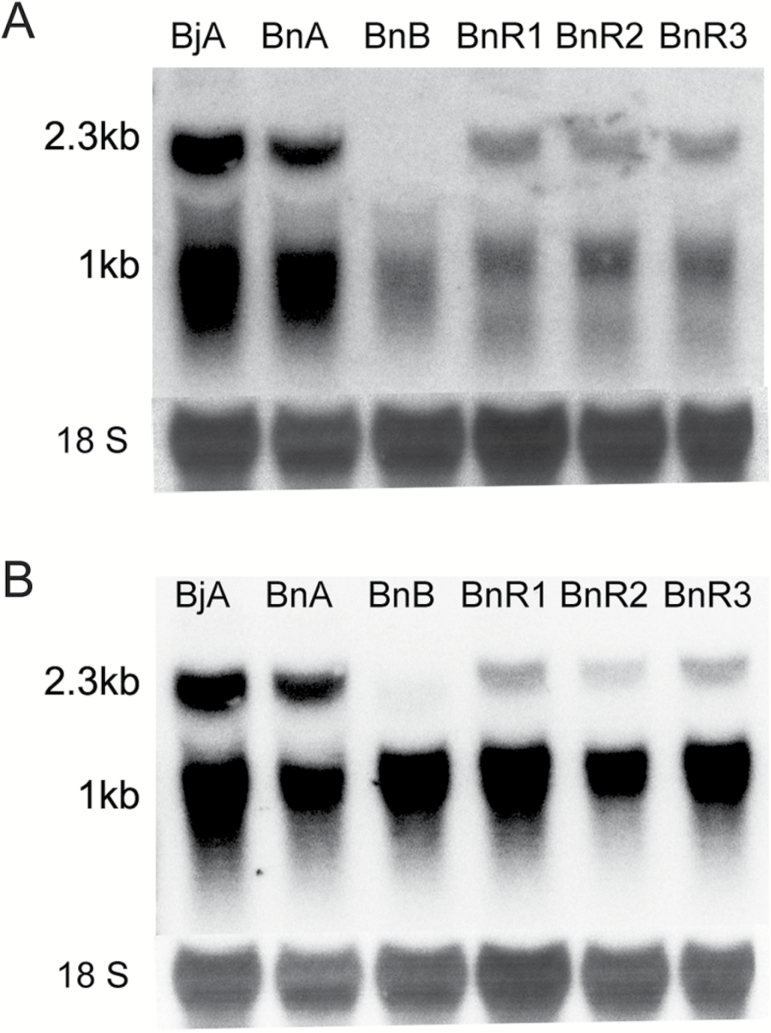
*orf288* and *atp6* expression patterns in the *hau* CMS lines. RNA gel-blotting analysis for (A) the *orf288* and (B) the *atp6* gene regions in the male-fertile, male-sterile, and nuclear-restorer floral buds: BjA, floral buds (<0.5 mm) from the *hau* CMS line (6-102A) in *B. juncea*; BjB, floral buds (<0.5 mm) from the *hau* CMS maintainer line (6-102B) in *B. juncea*; BnA, the male-sterile line in *B. napus*; and BnB, the maintainer line in *B. napus*. BnR1, BnR2 and BnR3 are different-sized floral buds (<0.5 mm; 0.5–2 mm; 2–4 mm) from the fertility-restorer line in *B. napus*.

The CMS-associated gene *orf288*, which is located downstream of and co-transcribed with *atp6*, is only expressed in the *hau* CMS line (Jing *et al.*, 2012). It was previously found to be constitutively expressed in all tested tissues of the *hau* CMS line, including flower buds, fresh leaves, roots, and hypocotyls (Heng *et al.*, 2014). Northern blotting was used to determine the *orf288* expression in different floral buds. Anthers from CMS *B. juncea* and *B. napus* plants and fertility-restorer *B. napus* plants showed both the 2.3-kb and 1-kb transcripts, but the intensity of both transcript bands was weak in fertility-restorer plants when the *orf288* fragment was used as a probe (Fig. 4A). When the fragment from *atp6* was used as a probe, the 2.3-kb and 1-kb transcripts were both present in the different lines, although the intensity of the 2.3-kb transcript band was weak in different floral buds of the *B. napus* fertility-restorer plants (Fig. 4B).

**Fig. 5.**
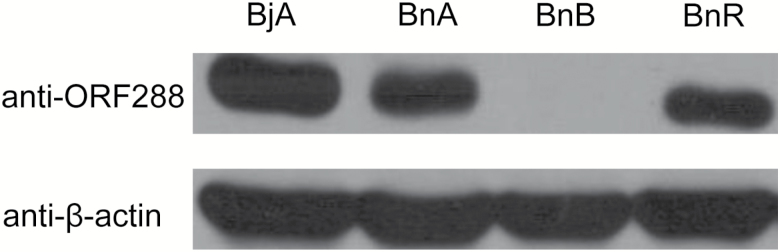
Western blotting analysis of ORF288 between the *hau* CMS lines and the fertility-restorer lines. BjA, floral buds (<0.5 mm) from the *hau* CMS line in *B. juncea*; BnA, floral buds (<0.5 mm) from the male-sterile line in *B. napus*; BnB, floral buds (<0.5 mm) from the maintainer line in *B. napus*; and BnR, floral buds (<0.5 mm) from the fertility restorer line in *B. napus*.

A western blotting assay was employed to further analyse ORF288 polypeptide expression levels in flower buds from the *B. juncea* and *B. napus* CMS lines and the *B. napus* fertility-restorer line. Intriguingly, equivalent amounts of ORF288 polypeptide were present in both the *hau* CMS line and the fertility-restorer line (Fig. 5).

### The cytotoxic region of the CMS-associated gene *orf288*

**Fig. 6.**
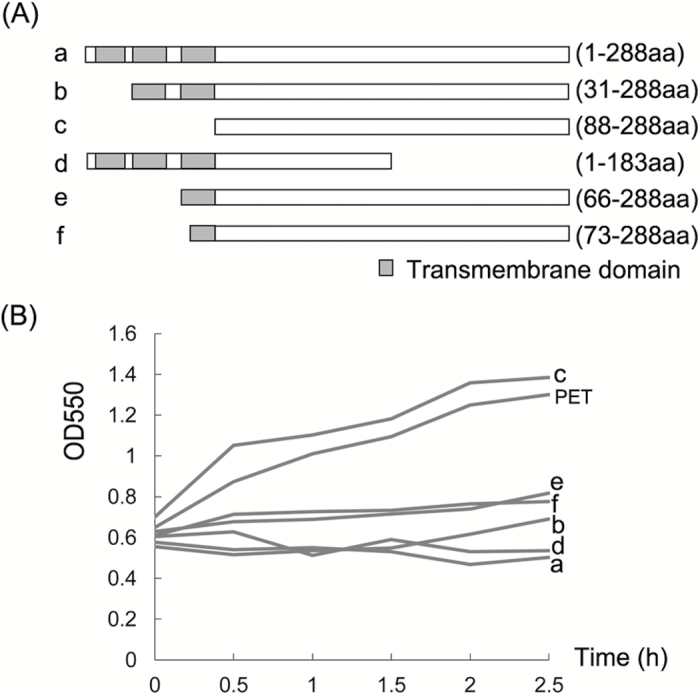
The cytotoxic region of the CMS-associated gene *orf288*. (A) Truncated *orf288* with different transmembrane domains: (a) full-length DNA sequence with three transmembrane domains; (b) truncated *orf288* without the first transmembrane domain; (c) truncated *orf288* without any transmembrane domains; (d) truncated *orf288* that does not include 105-aa at the C-terminus; (e) truncated *orf288* with only the third transmembrane domain; and (f) truncated *orf288* with only part of the third transmembrane domain. (B) Effect of the overexpression of different truncated *orf288* fragments on the growth of *E. coli* cells: (a–f) indicate the different truncated fragments shown in (A). IPTG was added when cell growth reached an OD_550_ of 0.6. PET indicates the control expression vector induced by IPTG.

Previous results using the software TMHMM server showed that ORF288 contains three transmembrane regions in its N-terminus (1–88 aa), and the CMS-associated gene *orf288* was found to significantly repress the growth of *E. coli* (Jing *et al.*, 2012). To further analyse the cytotoxic region in this CMS-associated gene, the full-length (Fig. 6Aa) and five truncated (Fig. 6Ab-f) versions of ORF288 were cloned and expressed using the pET32a vector. The empty PET vector without gene fragments was used as a control. The growth of *E. coli* was normal only when the cloned gene fragments did not include the three transmembrane domains (1–88 aa); a part or all of the ORF288 transmembrane domains inhibited *E. coli* growth (Fig. 6B). Thus, the toxicity region is contained in the three ORF288 transmembrane domains.

### A 215-aa region of the ORF288 C-terminus is sufficient to cause male sterility in Arabidopsis

**Fig. 7.**
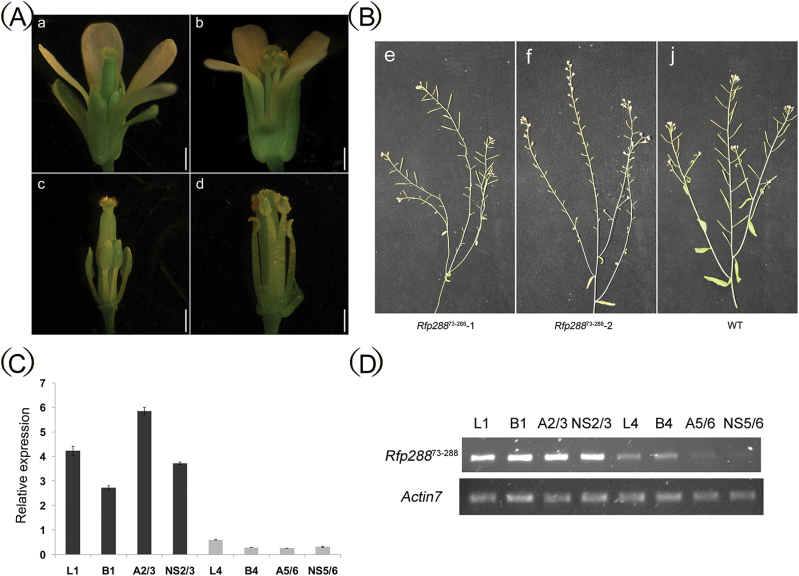
Fertility associated with truncated ORF288 expression in Arabidopsis. (A) Anthers from transgenic truncated *orf288* fragments (a, c) and the wild-type (b, d). (B) The siliques from transgenic truncated *orf288* fragments (e, f) and the wild-type (j). (C, D) qPCR and RT-PCR analysis of the expression levels of the truncated *orf288* fragments in different tissues from transgenic plants. L1 and B1, leaves and floral buds from a transgenic male sterility T_3_ plants; A2/3, anthers from two transgenic T_3_ male sterility plants; NS2/3, floral buds without anthers from two transgenic T_3_ male sterility plants; L4 and B4, leaves and floral buds from a transgenic semi-male sterility T_3_ plants; A5/6, anthers from two transgenic T_3_ semi-male sterility plants; and NS5/6, the floral buds without anthers from another two transgenic T_3_ semi-male sterility plants. (This figure is available in colour at *JXB* online.)

A previous study confirmed that *orf288* expression in Arabidopsis can lead to male sterility (Jing *et al.*, 2012). To further investigate which region of the CMS-associated *orf288* gene is responsible for male sterility, we generated five different transgenic *A. thaliana* lines containing either full-length ORF288, amino acids 1–88, or amino acids 73–288, with or without a mitochondria-targeting peptide, under a constitutive double CaMV35S promoter. Fourteen 2 × 35S::Rfp288, eighteen 2 × 35S::Rfp288^73–288^, nine 2 × 35S::288^73–288^, twenty 2 × 35S::Rfp288^1–88^, and eleven 2 × 35S::288^1–88^ (Fig. S1 A–D at Dryad) T_0_ plants were grown in the greenhouse for phenotypic evaluation. Full-length ORF288 or amino acids 73–288 of ORF288, with or without a mitochondria-targeting peptide, caused male sterility, whereas amino acids 1–88 of ORF288, with or without the mitochondria-targeting peptide, did not (Fig. 7A; Table S2 at Dryad). Among the 13 2 × 35S::Rfp288^73–288^ transgenic lines, most displayed total male sterility (Fig. 7Bf), and the anthers from a few plants displayed semi-sterility (Fig. 7Be). The 2 × 35S::Rfp288^73–288^ male sterility line was further crossed with WT pollen, and the *A. thaliana* T_3_ progeny plants could co-segregate with the stably introduced DNA. The line contained a single copy of 2 × 35S::Rfp288^73–288^ and showed a 1:1 segregation rate for the male sterility and fertility phenotypes. At the same time, the semi-sterile plants with 35S::Rfp288^73–288^ were self-seeded by generation. Plants expressing ORF288 amino acids 1–88, with or without the mitochondria-targeting peptide, did not show male sterility.

Quantitative PCR and real-time PCR with 2 × 35S:: Rfp288^73–288^-specific primers showed that Rfp288^73–288^ was relatively highly expressed in the leaves, floral buds, anthers, and floral buds without anthers in male-sterility T_3_ plants compared with semi-sterile T_3_ plants (Fig. 7C, D). Semi-thin sections were also used to compare anthers in the mature floral buds between WT and transgenic lines with 35S::Rfp288^73–288^. The anthers from the male-sterility flowers were completely abortive, and there were no pollen sacs or mature pollen (Fig. S2B at Dryad), distinct from the WT flowers (Fig. S2A at Dryad). However, the mature fertile anthers from the semi-sterile lines expressing 35S::Rfp288^73–288^ also showed abnormal development. Most anthers were affected to varying degrees, with some showing complete abortion and others showing only one or two pollen sacs with normal pollen grains (Fig. S2C, D at Dryad). Our transgenic analyses therefore confirmed that *orf288* is a key gene that can cause male sterility, and that it is the C-terminal region of ORF288 and not the N-terminal transmembrane domain that causes the sterility.

### Genes involved in early anther development are differentially expressed between the 6-102A and 6-102B lines

In this study, anther development was arrested during the archesporial cell differentiation stage in the *hau* CMS line. To better understand the processes associated with male anther sterility in this line, RNA was extracted from floral buds (<0.5 mm in length) of *hau* CMS plants and its iso-nuclear maintainer line and used to perform high-throughput transcriptome sequencing analysis (RNA-Seq). A total of 18 192 507 600 bp and 18 887 411 700 bp of clean data were separately generated with Q30 scores >92% from the *hau* CMS line and its iso-nuclear maintainer line, respectively (Table S3 at Dryad). The correlation coefficient (*R*^2^) between the different replicates calculated using RPKM values was greater than 0.95. In total, 5440 significant DEGs were identified between the *hau* CMS line and its iso-nuclear maintainer line (Table S4 at Dryad). Among these, 3256 unigenes were up-regulated and 2184 unigenes were down-regulated in the *hau* CMS line (Fig. S3 at Dryad).

**Fig. 8.**
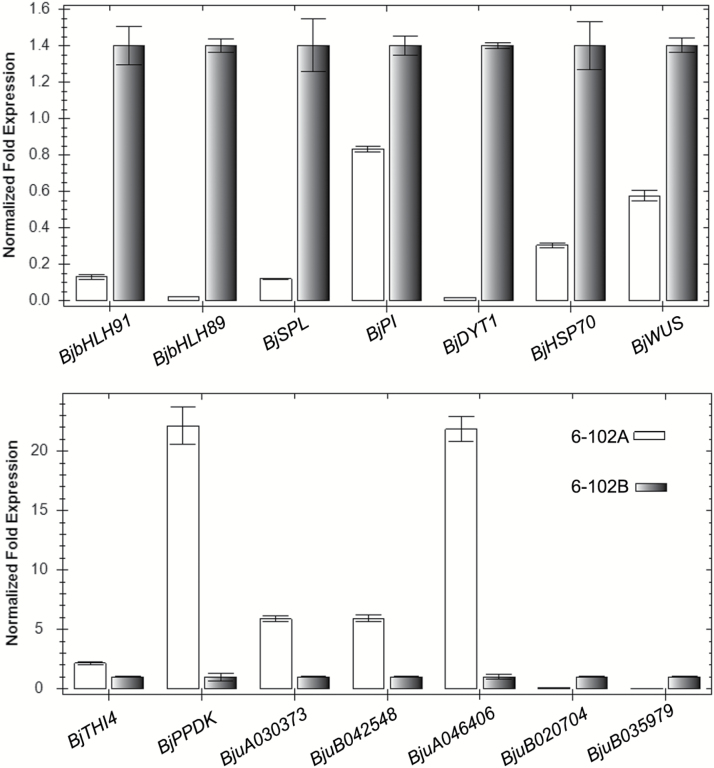
qRT-PCR analysis of the expression profile of homologous genes involved in early anther development in *B. juncea* for the *hau* CMS line (6-102A) and its maintainer line (6-102B). *Actin* was used as an internal control to normalize the transcript levels.

As the CMS phenotype is primarily caused by CMS genes located in the mitochondria, the expression profiles of DEGs in the mitochondria were further analysed. Genes involved in mitochondrial structure and ATP synthase were down-regulated in the *hau* CMS line (Table S5 at Dryad). Furthermore, genes involved in early anther development (e.g. *BjWUS*, *BjSPL*, *BjPI*, *BjDYT1*) were also down-regulated in anthers from the *hau* CMS line, whereas genes involved in autophagy, senescence, catalase, DNA repair, and mitochondrial DNA damage tolerance were up-regulated in this line (Table S5 at Dryad). In addition, a number of pentatricopeptide repeat (PPR) proteins were differentially expressed between the *hau* CMS and maintainer lines. qPCR results confirmed that these genes were differentially expressed between the *hau* CMS line and its maintainer line (Fig. 8). The *hau* CMS associated gene *orf288* may influence these DEGs in a retrograde manner, contributing to the *hau* CMS phenotype.

## Discussion

### Mitochondrial ultrastructure damage in anthers causes male sterility in *hau* CMS plants

In *Brassica*, numerous CMS-associated genes are expressed in the mitochondria and induce male sterility, although the specific anther abortive stages are distinct in different species. In *B. napus*, the *pol* CMS anther produces little or no pollen and shows no differentiation of sporogenous cells (An *et al.*, 2014; Liu *et al.*, 2016). The abortive stage of *ogu* INRA CMS in rapeseed begins as early as the tetrad stage (González-Melendi *et al.*, 2008; Yang *et al.*, 2008), although petaloid stamens were observed in *ogu* CMS in *B. juncea* (Kirti *et al.*, 1995; Meur *et al.*, 2006). The abortive stages and detailed subcellular events in anther development in many different CMS plants have been analysed by light and electron microscopy (Chen and Liu, 2014). Mitochondrial structure in tapetal cells that developed within sterile anthers was found to be greatly affected compared with the WT in *ogu* INRA CMS (González-Melendi *et al.*, 2008). Mitochondria from the tapetum cells in engineered male-sterile tobacco plants lost their cristae and appeared swollen (Hernould *et al.*, 1998). Furthermore, tapetal cells became distorted when mitochondrial aberrations were present in *Petunia hybrida* (Bino, 1985). High amounts of hydrogen peroxide around the mitochondrial outer membranes were detected in the rice ZS97A tapetum at the microspore mother cell (MMC) stage, but not in ZS97B or in the later stage tapetum cells from ZS97A (Luo *et al.*, 2013). As in other CMS plants, ultrastructural changes in mitochondria from *hau* CMS systems affected normal mitochondrial function, arrested archesporial cell development, and eventually caused male sterility. We speculate that male sterility in the *hau* CMS line is primarily due to mitochondrial dysfunction in the archesporial cells, which arrests cell development.

### Expression levels of the CMS-associated gene *orf288* affect the degree of male sterility

By comparing CMS lines and their fertility-restorer lines, we can better understand the expression profiles of CMS-associated genes. For example, the *nap* CMS-associated gene *orf222* blocks pollen development at specific early stages. The nuclear *Rf* gene reduces *orf222* transcript levels in a tissue-specific manner (Geddy *et al.*, 2005). The *pol* CMS restorer-fertility gene *Rfp* acts to post-transcriptionally decrease *orf224* transcript levels (Menassa *et al.*, 1999; Liu *et al.*, 2016). *PPR-B*, which is the *ogu* CMS *Rf* gene, does not affect the local accumulation of *orf138* mRNA in young anthers, but it does inhibit ORF138 synthesis in the tapetum (Uyttewaal *et al.*, 2008). Transcript levels of the CMS-associated gene *orf108* were found to be reduced by the *Rf* gene in *B. juncea* containing *Moricandia arvensis* cytoplasm (Ashutosh *et al.*, 2008; Kumar *et al.*, 2012). Northern blotting analysis revealed that transcripts for the *hau* CMS-associated gene *orf288* were present at higher levels in *hau* CMS flower buds than in the restorer line. However, the protein levels of ORF288 displayed no differences between the *hau* CMS line and its fertility-restorer line. Although maize *Rf2* can restore fertility to T cytoplasm plants, it does not alter the accumulation of URF13 (Liu *et al.*, 2001). In the I-12CMS(3) cytoplasm from wild beet, *orf129* is responsible for male sterility, although the restorer genes do not affect ORF129 protein accumulation (Yamamoto *et al.*, 2008). Our results suggest that *Rf* genes suppress *atp6/orf288* through a post-translational mechanism to restore male fertility. In our transformation experiment, we placed different truncated *orf288* fragments, with or without the mitochondria-targeting peptide, under the double CamV35S promoter and introduced them into Arabidopsis. Amino acids 73–288 from ORF288 induced male sterility, whereas amino acids 1–88 did not. In rice WA CMS, different truncated fragments of the WA CMS-associated gene *WA352* were transformed into their maintainer line, and transgenic plants with MTS-WA352_218–300_ and MTS-WA352_282–352_ were male-sterile, whereas those with MTS-WA352_1–227_ were fertile (Luo *et al.*, 2013); therefore, it was concluded that amino acids 218–352 were the core region that caused male sterility. The *orf288* expression levels in different transgenic lines determined the anther abortive stage. The *hau* CMS cytoplasm was also transformed into *B. napus*: most of the plants were completely male-sterile, although some were not completely abortive in certain nuclear backgrounds. In this line, only one or two anthers had pollen grains; therefore, some minor restorative genes may influence the expression of *orf288* in these plants. These results further indicate that *orf288* transcript levels influence the degree of male fertility.

### The cytotoxic region of ORF288 is not associated with cytoplasmic male sterility

The *hau* CMS-associated protein ORF288, which significantly represses *E. coli* growth, is toxic to the host cells (Jing *et al.*, 2012); however, the identity of the regions that cause cytotoxicity and male sterility were unknown. In our study, various truncated fragments of *orf288* with or without the transmembrane domains were cloned into an inducible vector and expressed using IPTG in *E. coli*. Interestingly, only the fragment with the transmembrane domain was cytotoxic to *E. coli* growth (Fig. 6B). This suggests that amino acids 1–88 of ORF288, which contain the three transmembrane domains, are the cytotoxic region. In rice, the WA CMS-associated protein WA352 has also been shown to be toxic to *E. coli*, although a truncated WA352 protein that did not contain the cytotoxic region still caused male sterility when expressed in transgenic plants (Chen and Liu, 2014). To better understand whether ORF288 cytotoxicity influences male sterility, we transformed amino acids 1–88 from ORF288, with or without a mitochondria-targeting peptide, into Arabidopsis, and found that they did not cause male sterility. But amino acids 73–288 from ORF288 with or without a mitochondria-targeting peptide did cause male sterility. The subcellular localization of ORF288^73–288^ was to mitochondria, as predicted by PredSL (http://aias.biol.uoa.gr/PredSL/;Petsalaki *et al.*, 2006). These results may illustrate why the transformation with 2 × 35S::288^73–288^ lacking the mitochondrial targeting peptide could produce male sterility. A number of CMS-associated proteins in other crop plants have also been shown to be toxic to *E. coli*, such as URF13 in maize CMS-T (Korth *et al.*, 1991), ORF522 in sunflower CMS-PET1 (Nakai *et al.*, 1995), ORF138 in radish CMS-Ogu (Duroc *et al.*, 2005), and ORF79 in rice CMS-BT (Wang *et al.*, 2006). However, direct evidence of protein cytotoxicity causing male sterility is still lacking. Indeed, here we found that the cytotoxic regions in the *hau* CMS gene *orf288* do not cause male sterility.

### *orf288* may control nuclear genes through retrograde regulation

High-throughput sequencing approaches have been used to reveal the genetic regulatory networks underlying early anther development by comparative gene expression analysis between mutant and WT plants. Different bioprocesses and genes involved in energy deficiency and early anther development are down-regulated in the *pol* CMS anther through nuclear–mitochondrial interactions (An *et al.*, 2014). In *B. juncea orf220*-type CMS, some genes related to mitochondrial energy metabolism and pollen development were found to be down-regulated in transgenic plants (Yang *et al.*, 2010). MSH1-RNAi lines with increased copy numbers of ORF220 caused male sterility, and numerous genes involved in anther development were up- or down-regulated in revertant and MSH1-RNAi lines (Zhao *et al.*, 2016). In our study, genes involved in mitochondrial structure, ATP synthase, and early anther development were down-regulated in the *hau* CMS line, whilst genes involved in autophagy, senescence, catalase, DNA repair, and mitochondrial DNA damage tolerance were up-regulated in anthers from this line. These differentially expressed PPR genes may be responsible for many different post-transcriptional events, such as RNA editing and processing. These DEGs could affect archesporial cell differentiation and induce male sterility. The results suggest that CMS genes located in the mitochondria indirectly affect nuclear genes through retrograde regulation. Comparative analysis of these DEGs could provide a comprehensive understanding of the mechanism underlying *hau* CMS in *B. juncea*. Cytoplasmic male sterility promotes outcrosses and increases hybrid seed production in *Brassica*. However, exactly how the *hau* CMS-associated mitochondrial gene *orf288* arrests anther development during the archesporial cell differentiation stage requires further study. Better characterization of the *hau* CMS phenotype should help to unravel the mechanism of male sterility in *Brassica*.

## Data deposition

The following figures and tables are available at Dryad Data

Repository: https://doi.org/10.5061/dryad.9s68p.

Fig. S1. Schematic showing truncated *orf288* gene construction.

Fig. S2. Semi-thin sections from transgenic *Arabidopsis* expressing truncated *orf288*.

Table S1. Primers used in this study.

Table S2. Fertility statistics for the mitochondria-targeted expression of the truncated *orf288* fragment in *Arabidopsis thaliana*.

Table S3. Summary of RNA-Seq data in 6-102A and 6-102B.

Table S4. Detailed information on the differentially expressed genes between 6-102A and 6-102B.

Table S5. Selected differentially expressed genes.
